# Topical ambroxol for the treatment of neuropathic pain: An initial clinical observation. English version

**DOI:** 10.1007/s00482-015-0060-y

**Published:** 2015-11-20

**Authors:** K.-U. Kern, T. Weiser

**Affiliations:** 1 Institut für Schmerzmedizin/Schmerzpraxis Wiesbaden, Sonnenberger Str. 68, 65193 Wiesbaden, Germany; 2 Boehringer Ingelheim Pharma GmbH & Co. KG, Ingelheim, Germany

**Keywords:** Ambroxol, Neuropathic pain, Topical therapy, Na_v_ 1.8, Local anaesthetic, Ambroxol, Neuropathischer Schmerz, Topische Therapie, Na_v_ 1.8, Lokalanästhesie

## Abstract

**Background:**

Neuropathic pain is difficult to treat, and the available options are often inadequate. The expectorant ambroxol also acts as a strong local anaesthetic and blocks sodium channels about 40 times more potently than lidocaine. It preferentially inhibits the channel subtype Na_v_ 1.8, which is expressed especially in nociceptive C-fibres. In view of the low toxicity of ambroxol, it seemed reasonable to try using it for the treatment of neuropathic pain that failed to respond to other standard options.

**Material and methods:**

The medical records of seven patients with severe neuropathic pain and pain reduction following topical ambroxol treatment are reported retrospectively. As standard therapies had not proved sufficient, a topical ambroxol 20 % cream was repeatedly applied by the patients in the area of neuropathic pain.

**Results:**

The reasons for neuropathic pain were postherpetic neuralgia (2 ×), mononeuropathy multiplex, phantom pain, deafferentation pain, postoperative neuralgia and foot neuropathy of unknown origin. The individual mean pain intensity reported was between 4 and 6/10 (NRS), maximum pain at 6–10/10 (NRS). The pain reduction achieved individually following ambroxol cream was 2–8 points (NRS) within 5–30 min and lasted for 3–8 h. Pain attacks were reduced in all five patients presenting with this problem. Four patients with no improvement after lidocaine 5 % and one patient with no response to capsaicin 8 % nevertheless experienced a pain reduction with topical ambroxol. No patient reported any side effects or skin changes during a treatment that has since been continued for up to 4 years.

**Conclusion:**

Ambroxol acts as a strong local anaesthetic and preferentially inhibits the nociceptively relevant sodium channel subtype Na_v_ 1.8. For the first time, we report below on a relevant pain relief following topical ambroxol 20 % cream in patients with neuropathic pain. In view of the positive side effect profile, the clinical benefit in patients with pain should be investigated further.

## Abstract (de)

**Hintergrund:**

Neuropathische Schmerzen sind schwer behandelbar, die vorhandenen Therapien oft nicht ausreichend. Das Sekretolytikum Ambroxol hat auch stark lokalanästhetische Eigenschaften und hemmt Natriumkanäle ca. 40 mal potenter als Lidocain. Es blockiert bevorzugt den Kanalsubtyp Na_v_ 1.8, der besonders in nozizeptiven C-Fasern exprimiert wird. Bei geringer Toxizität erschien ein Behandlungsversuch mit topischen Ambroxol berechtigt bei unzureichend behandelbaren, neuropathischen Schmerzen.

**Material und Methode:**

Retrospektiv werden exemplarisch 7 Krankheitsverläufe von Patienten mit schweren, neuropathischenSchmerzen dargestellt, bei denen viele Behandlungsversuche mit zugelassenen Substanzen erfolglos odernicht möglich waren. Diese waren in einem umschriebenen Areal topisch mit einer Ambroxol 20 %-Cremebehandelt worden.

**Ergebnisse:**

Schmerzursachen waren: Postzosterneuralgie (2x), Mononeuropathia multiplex, Deafferenzierungsschmerz, Phantomschmerz, postoperative Neuralgie und unklare Fußneuropathie. Diedurchschnittlichen Schmerzstärken lagen zwischen 4–6/10 (NRS), maximal bei 6–10/10. DieSchmerzreduktionen unter Ambroxol betrugen 2–8 Punkte (NRS) binnen 15–30 min und hielten 3–8 h an.Schmerzattacken reduzierten sich bei allen 5 Betroffenen. 4 Patienten ohne vorherige Besserung auf Lidocain 5 % und 1 Patient ohne Linderung auf Capsaicin 8% profitierten dennoch von topischem Ambroxol. Kein Patient berichtete Nebenwirkungen oder Hautveränderungen, die längste Behandlung überblickt mittlerweile 4 Jahre.

**Schlussfolgerung:**

Ambroxol wirkt als starkes Lokalanästhetikum und blockiert bevorzugt den nozizeptiv relevanten Kanalsubtyp Na_v_ 1.8. Wir berichten hier erstmals über eine relevante Schmerzlinderung durch topisches Ambroxol 20 % bei Patienten mit neuropathischem Schmerz. Angesichts des günstigen Nebenwirkungsprofils sollte der klinische Nutzen für Schmerzpatienten dringend weiter untersucht werden.

## Background and problem

Neuropathic pain is common and difficult to treat. However, topical treatments such as transient functional desensitization of TRPV1 receptors as well as C-fibres with capsaicin 8 % and blockade of neuronal signal transmission with lidocaine 5 % are often inadequate. Other potent inhibitors of cutaneous nociceptors with few side effects would be welcome as topical therapeutic options.

Ambroxol has been authorized for the treatment of respiratory disorders since 1979 and can now be freely purchased over the counter. Since its local anaesthetic properties were recognized at an early stage [23], ambroxol-containing pastilles have also been authorized for the treatment of sore throat [10]. However, the substance has never been used to date as an analgesic, although pain-related behaviours have been suppressed in animal studies, even in chronic pain situations [14, 16, 27, 32]. Compared with local anaesthetics, ambroxol is, interestingly, a very potent blocker of voltage-dependent sodium channels, blocking these channels about 40 times more strongly than lidocaine [47]. Moreover, the sodium channel subtype Na_v_ 1.8, which is preferentially expressed in nociceptive C-fibre neurons [2, 6, 35, 52], is blocked more potently compared to the other channel subtypes. Since the toxicity of the substances is comparatively very low [51, 53], the use of an ambroxol-containing semisolid, topical dosage form may represent an interesting approach to the treatment of what are otherwise difficult to treat pain conditions.

We report below on a clinically relevant analgesia produced by topical ambroxol in neuropathic pain.

### Method

By way of example, we present the case histories of seven patients with neuropathic pain that was resistant to other/standard treatment and who benefitted from topical ambroxol. Within the setting of an outpatient pain clinic they were treated in a peripheral, defined part of the body after numerous other therapeutic attempts with authorized substances had proved unsuccessful or impossible. The preparation used was a 20 % cream (ambroxol cream 20 %, 50.0 g: ambroxol 10.0 g, dimethyl sulfoxide 5.0 g, made up to 50.0 g with Linola cream), which was prepared pharmaceutically for each individual patient. In tests this formulation was shown to possess the highest concentration of still soluble ambroxol.

While test treatments were administered during the consultation, in the majority of cases the observations reported here were collected by the patients after they themselves had applied the preparation to the painful area over a prolonged period at home. Clinical observations and reports were documented in the medical records and retrospectively served as the basis for the case reports.

All the patients gave their written informed consent both to the individual test treatment with ambroxol-containing creams (which were prepared individually and are not available as proprietary preparations) and also to the use of the anonymized reports, findings and possible images/videos. The observations presented here by way of example refer to a period of 4 years overall and were documented and reported on the basis of the German professional code of conduct for doctors (particularly §15) and the requirements of the Declaration of Helsinki (§37). The treatments were prescribed outside the health insurance reimbursement scheme.

## Results

We report the clinical observations collected for seven patients, two women and five men. The causes of their neuropathic pain were as follows: postherpetic neuralgia (2 ×), mononeuropathy multiplex (1 ×), postoperative neuralgia (1 ×), deafferentation pain (1 ×), phantom pain (1 ×) and foot neuropathy of unknown origin (1 ×). The average pain intensity reported by the patients was between 4/10 and 6/10 (NRS), while the maximum pain intensity was between 6/10 and 10/10 (NRS). The pain reduction achieved was between 2 and 8 points (NRS). The patients observed a reduction in pain within 5–30 min, which lasted for between 3 and > 8 h (Tab. 1). Five patients additionally suffered from pain attacks, and these were reduced in all cases. Four of the seven patients noticed clear functional improvements (physical activity, increased mobility, improved sleep, ability to work, etc.). None of the patients reported side effects or, more particularly, skin changes. None of the patients had been receiving this treatment as their sole medication, and all continued taking primary medication, with occasionally varying dosages, over what was a prolonged treatment period in some cases. The patient with the longest treatment period has been using topical ambroxol for 48 months (patient 1), while the shortest case report (patient 4) covers a period of 9 months (Table 2).

**Table 1:** Diagnoses and pain characteristics of treated patients

Case	Diagnosis of neuropathic pain	Duration of pain (months)	NRS		Allodynia		Effects of topical application	
Nr.			Mean	Max	Dynamic	Pin-prick	Lidocaine 5 %	Capsaicin 8 %
1	Neuropathic forefoot pain	55	5	8	Yes	Yes	No	Yes
2	Cold phantom pain	68	6	8	Yes	No	No	n.t.
3	Neuropathic pain after total knee replacement	49	6	10	Yes	Yes	No	Yes
4	Deafferentation pain	174	4	8	Yes	Yes	No	n.t.
5	Thoracic postherpetic neuralgia	78	4	6	Yes	No	Yes	No
6	Mononeuropathy multiplex	108	4	8	No	No	No	n.t.
7	Trigeminal postherpetic neuralgia	6	5	8	Yes	Yes	Yes	n.t.

*NRS* numerical rating scale, *n.t.* not tested

**Table 2:** Pain relief by topical ambroxol. Time of usage, functional improvement and adverse drug reactions (ADR)

Fall	Diagnosis	Ambroxol applied since (months)	Relief of	ADR
1	Neuropathic forefoot pain	43	Walking, gardening	No
2	Cold phantom pain	10	Sleep	No
3	Neuropathic pain after total knee replacement	7	Movement	No
4	Deafferentation pain	4	Continuing work	No
5	Thoracic postherpetic neuralgia	32	Sleep	No
6	Mononeuropathy multiplex	12	Watching TV, sleep	No
7	Trigeminal postherpetic neuralgia	5	Sleep	No

### Allodynia

Dynamic allodynia was present in six of the seven patients and a hyperalgesia in response to pinprick stimuli in four patients. Both forms were present in four cases, while one patient suffered from neither. Dynamic allodynia on its own was observed in two cases, and no patient was sensitive exclusively to pinprick stimuli (Table 1). Irrespective of these configurations, all patients were sensitive to ambroxol.

### Pretreatment with lidocaine 5 %

Six of the seven patients had previously used lidocaine plasters, which proved ineffective in four cases and helpful in two patients, one of whom was unable to tolerate them. Both of the patients experiencing pain relief also reported this with ambroxol. By contrast however, all four patients in whom the lidocaine had proved ineffective profited additionally from the effect of ambroxol.

### Pretreatment with capsaicin 8 %

Three of the seven patients had also received capsaicin 8 % as topical treatment. In two patients this was helpful, and one continues to use the medication. Both patients whose pain was relieved by capsaicin 8 % also experienced this relief with ambroxol. By contrast however, the patient in whom the capsaicin had proved ineffective benefitted from topical ambroxol.

## Case reports

### Case report 1 (Neuropathic forefoot pain)

Born in 1942, this man presented in October 2010 (clinically) with neuropathic pain in both feet. He felt as if he was running on coals, while his right foot felt as if it were gripped in a vice. Walking or gardening was almost impossible. The foot pain could not be explained in orthopaedic terms, either on the basis of radiographs or as a result of a spondylodesis of L4/5 in 2007, and no polyneuropathy was detected on neurographic investigation. The problem was thought to be ‘likely of vertebral origin’. Clinical examination showed pronounced dynamic allodynia and hyperalgesia in response to pinprick stimuli in the arch of the right foot, and to a lesser extent in the left foot. Gabapentin had been prescribed up to a dosage of 3 × 600 mg, supplemented by buprenorphine 20 µg/h, and no further dose increase were possible in either case. Topical lidocaine 5 % plasters had not proved helpful.

**Fig. 1:**
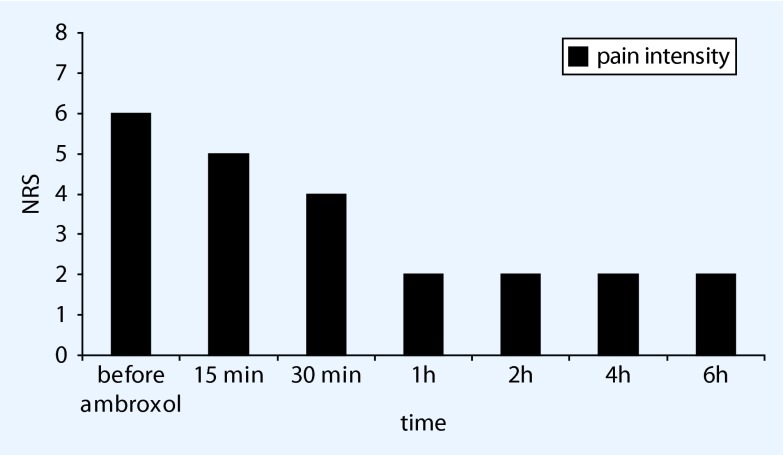
Pain reduction following topical ambroxol 20 % in neuropathic forefoot pain (Exemplary documentation of patient 1, *NRS* numerical rating scale)

The first test treatment with topical ambroxol was started in June 2011, with a very successful outcome: within 5 min the stabbing pain, with an initial intensity of 8/10 on the NRS scale during the heel-to-toe roll of the right foot, and the touch sensitivity of the forefoot disappeared completely for over 8 h. The intermittent treatment with topical ambroxol has been continued to date successfully (Fig. 1). The pain has also meanwhile been treated successfully on 11 occasions with capsaicin 8 % plasters, and the patient now uses ambroxol cream in phases when the pain recurs and the capsaicin effect is wearing off prior to the next application. He now takes gabapentin only in the evening at a dosage of 300 mg, while the opiate has been discontinued. Walking and gardening are now possible again. No skin reactions or other side effects have occurred during the treatment that has proved effective till now, without any changes, for 4 years. This case report has been documented repeatedly on video.

### Case report 2 (Cold phantom pain)

This patient presented with unusual pains: he complained of extremely painful cold sensations in both previously amputated phantom feet. A below-knee amputation of the left leg had been required for pAOD and diabetes mellitus in 2008, followed by the right leg in 2009. The cold pains, with an intensity of NRS 7–9/10, occurred sporadically several times a week, lasted from a few minutes to many hours and frequently woke the patient at night. He described the cold sensation as shifting between the toes and balls of the feet and said that heat applied to the stump was of limited benefit. The cold phantom pain could be triggered by a cold environment, but also by visually perceived cold (e.g. images of snow). He had not experienced the cold sensation prior to amputation (in terms of preamputation recall), and the stump itself was usually warm. Both stumps showed slightly increased cold perception on the distal lateral sides, on the right with additional dynamic allodynia (without pinprick hyperalgesia). Following many unsuccessful treatments (includes opiates and anticonvulsants), the topical ambroxol 20 % cream managed to produce a relevant effect: the extreme cold sensation in the phantom regressed distinctly after approximately 15 min for several hours, and the phantom limb felt warmer. This effect has persisted unchanged for over 11 months now. No skin changes of any kind or any other side effects occurred on the stump, and the case report is documented on video.

### Case report 3 (Neuropathic pain after total knee replacement)

**Fig. 2:**
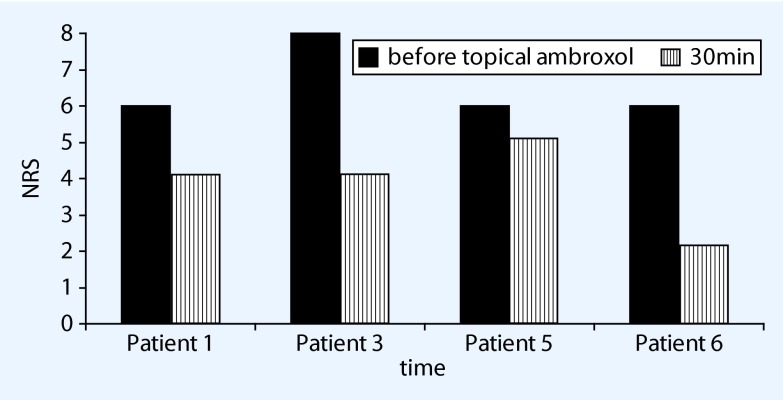
Early pain reduction, 30 min after topical ambroxol 20 % (exemplary documentation of some patients, *NRS* numerical rating scale)

This 58-year-old female patient received a total knee replacement in November 2010, after which considerable pains persisted. Clinical examination revealed an extensive dynamic allodynia and pinprick hyperalgesia medially as a sign of central sensitization. There were no signs of inflammation or movement-related pain. The existing treatment with buprenorphine 10 ug/h had been replaced by tapentadol because of the neuropathic nature of the pain, but this had proved no better in relieving the pain. After capsaicin 8 % plasters, the pain became slightly less frequent and of shorter duration. Since the knee pain was still persisting substantially by April 2014 and lidocaine plasters had also not been very helpful, topical ambroxol 20 % was used during the consultation. After just 15 min the patient reported clear pain relief: the burning and stabbing had subsided distinctly, while a ‘*raging feeling*’ in the knee disappeared almost completely. After applying the cream repeatedly over the next few months, she observed a reduction in pain for 4–6 h after approximately 30 min in each case, from an average of NRS 8/10 before treatment to 4/10 NRS (Fig. 2), and occasionally even down to NRS 1/10. With the most extreme pain (NRS 10/10), she found that the pain no longer returned to this level after the treatment. She has been applying topical ambroxol 20 % cream regularly for 11 months now, without any side effects or skin reactions. The case report is documented on video.

### Case report 4 (Deafferentation pain)

The 38-year-old patient presented in April 2014 with deafferentation pains in the left arm after a plexus lesion (motorcycle accident in 1997), with subsequent mechanical allodynia in hand and forearm. A nerve graft proved unsuccessful, as did ketamine, gabapentin and a lidocaine infusion. Mirror therapy ended with a worsening of the pain for several days, while amitriptyline produced excessive sedation. Although cannabis (in connection with a clinical study) managed to reduce the pain by 60 %, it also resulted in substantial mental impairment. His medication now consisted of pregabalin 2 × 300 mg and duloxetine 60 mg. Slight innervation of the biceps muscle and shoulder elevation were possible, as was mental motor imagery to modulate the phantom pain. The patient experienced three types of pain in the arm: a ‘*burning pain*’ (started by the application of cold to the hand), a ‘*crushing underlying pain*’ (with no trigger) and ‘*shooting tingling pains*’. The pain intensity ranged from NRS 4–8/10, and there was no dynamic allodynia at the elbow. Sensations could be triggered in the phantom arm by cold stimuli to the subclavicular area and, as referred pains, from trigger points from the subclavian and pectoral muscles. Lidocaine plasters merely changed the nature of the pain, while trigger point treatments and tapentadol were not tolerated. A test was therefore initiated with topical ambroxol 20 % cream over the pectoral muscle, which successfully relieved the shooting and tingling pains in most cases from NRS 8/10 to 4/10. Since then the effect has been described as starting after approximately 15 min and persisting for 4–6 h, which proved crucially important particularly in falling asleep. However, the ‘*deep underlying pain*’ remained unaffected. The patient was especially happy about the spontaneous cessation, without triggers, of repeatedly occurring spasms and cramps, which enabled him to continue everyday tasks at these times. The patient’s descriptions are documented on video.

### Case report 5 (Thoracic postherpetic neuralgia)

**Fig. 3:**
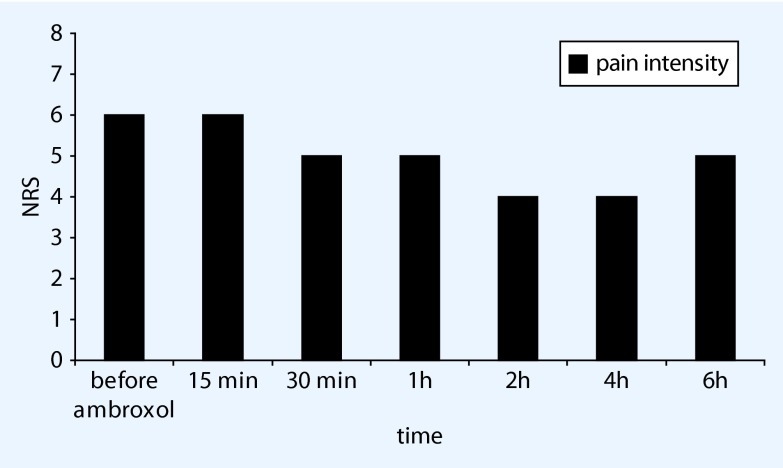
Pain reduction following topical ambroxol 20 % in postherpetic neuralgia (exemplary documentation of patient 5, *NRS* numerical rating scale)

This 55-year-old patient presented in August 2008 with a postherpetic neuralgia on the right side of the chest (intensity 5/10) that had been present for 2 months. Step I and Step II analgesics only reduced the pain slightly, while lidocaine plasters proved very effective. Clinical examination revealed an almost circular, dynamic allodynia at T6-8 on the right. He was initially treated successfully with increasing doses of gabapentin, and his night-time sleep improved with amitriptyline drops. Since other sedating substances were not possible and capsaicin 8 % remained unsuccessful, the use of topical ambroxol 20 % cream was tried in April 2012. Although this was unable to control the pain adequately on its own, it did manage to reduce the pain, starting after 30 min, from NRS 6/10 to 4/10 (Fig. 3) for a period of 4–6 h. The patient applies the cream for pain attacks and for those areas that are not well covered by the lidocaine plasters, which he continues to use. No adverse effects or skin reactions have been reported after what is now more than 3 years of use.

### Case report 6 (mononeuropathy multiplex)

**Fig. 4:**
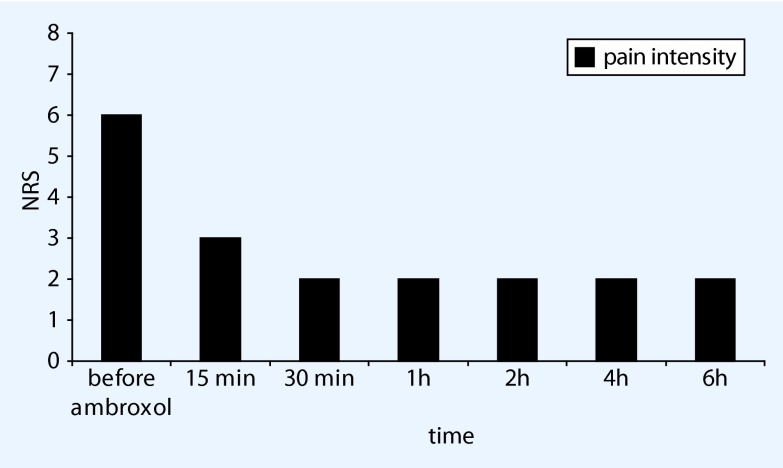
Pain reduction following topical ambroxol 20 % in mononeuropathia multiplex, (exemplary documentation of patient 6, *NRS* numerical rating scale)

This patient presented in November 2013 with neuropathic pain in the arch of the left foot and multiplex neuritis as a result of vasculitis. He described two types of pain: a permanent underlying pain that interfered considerably with this everyday activities, and he also reported attacks of fierce pain, particularly in the evenings while watching television and at night, with an intensity up to NRS 8/10. Meanwhile he has been receiving treatment for depression with duloxetine 60 mg and lithium. Lidocaine plasters, peripheral analgesics and low-dose tilidine had already been tried without success. His night-time sleep was now deepened with amitriptyline drops, while the dosage of the tilidine was cautiously increased. Since the central nervous tolerability threshold had already been reached at the second visit in December 2013, a test was arranged with topical ambroxol. This proved highly effective: the patient reported a reduction in his evening, neuropathic pain from NRS 6/10 to 2/10, starting after 15 min and lasting for over 6 h. He was able to watch television without interruption, while his night-time sleep (after applying the cream immediately beforehand) was usually good. The zolpidem that he had been using for a long time was now unnecessary. When he was woken by pain, further application of the cream produced rapid pain relief. After 4 months of use, the permanent underlying pain during the day disappeared almost completely. A notable finding was the absence before treatment in this patient of any kind of allodynia or hyperalgesia in response to dynamic or pinprick stimuli or to cold or draughts of air. Having applied the cream for a period of 17 months now, the patient continues to use this treatment (Fig. 4) and has not reported any form of skin reactions or side effects to date. The patient’s statements have been documented repeatedly on video.

### Case report 7 (Trigeminal postherpetic neuralgia)

After contracting a zoster infection of the V2 branch on the left in June 2014, this 91-year-old female complained of facial pain up to the intensity of NRS 8/10. Affective pain perception was considerable, and her night-time sleep was greatly disturbed. Diclofenac, tramadol and metamizole were not sufficient. Her night-time sleep was initially improved with a few amitriptyline drops, but an increase in the tramadol dosage failed due to constipation and sedation. Lidocaine plasters were effective, but had to be discontinued due to skin reactions. Since the dosage of gabapentin (100 mg) could only be increased to a limited extent and capsaicin 8 % did not appear to be indicated in this elderly woman, ambroxol 20 % cream was attempted. She has now been using this for 11 months and describes the effect as clearly pain-relieving, and felt that her cheek became ‘calmer’ approximately 15 min after each application. If she awoke at night, she was able to fall asleep again without pain sometime after a further application. This use has also been continued now for 10 months without any adverse effects and is documented on video.

## Discussion

In this case series we describe, for the first time, patients whose neuropathic pain was relieved by the topical application of ambroxol. The pain reduction was between 33 and 100 % (or between 2 and 8 NRS points; Table 1) and can therefore be considered as clinically relevant [12]. The findings can be explained by the sodium channel blockade produced by the substance.

### Pharmacology, sodium channels and analgesic effect

Ambroxol is structurally similar to known local anaesthetics (‘Löfgren structure’) and, like these, binds to a specific binding site of the neuronal sodium channel [27]. It likewise blocks the sodium influx at voltage-dependent sodium channels, which leads to a reduction in the action potential frequency and thus in intraneural signal transduction.

However, certain properties suggest that ambroxol is of particular interest for the treatment of neuropathic pain. Firstly, it is very potent: ambroxol inhibits neuronal sodium channels at an approximately 40-fold lower concentration than lidocaine [47], thereby alleviating neuropathic pain after local [29] and systemic application [13]. Secondly, in animal studies (and in contrast with lidocaine), it blocks nociceptor-specific sodium channels more potently than other neuronal sodium channels [47]. This may explain why four of our patients in whom lidocaine had previously proved ineffective nevertheless profited from ambroxol, which also produced a very powerful effect in animal models of neuropathic pain after systemic administration [14, 16], at least as strong as that of gabapentin and at dosages that can certainly be employed in clinical practice [14, 49].

Voltage-gated sodium channels (Na_v_; v = voltage gated) in nerve cell membranes play an important role in signal transduction, including in nociceptive neurons. Nine subtypes are described (Na_v_ 1.1–1.9) [8]. The neuronally expressed channels Na_v_ 1.1, 1.2, 1.3, 1.6 and 1.7 are inhibited with high affinity by tetrodotoxin (TTX), the toxin of the pufferfish. The subtypes expressed predominantly in nociceptive neurons, that is Na_v_ 1.8 and 1.9, are TTX-resistant [37]. Ambroxol blocks TTX-resistant sodium channels to a much greater degree than TTX-sensitive channels. The TTX-r-subtype Na_v_ 1.8, which is preferentially blocked by ambroxol, is expressed particularly in spinal ganglion cells [2]. In nerve injuries, it appears to play an important role in the sensitization of nociceptors and thus contribute to the development and maintenance of neuropathic pain [3, 21, 26, 36]. It is thought to be particularly important in pain caused by cold [52]. A connection with the observation of ‘warming of the ice-cold phantom leg’ by the application of ambroxol to the stump of patient 2 remains to be seen. Na_v_ 1.8 blockade also proved to be clearly analgesic in animal studies [11, 20]. So the improvements in our patients are credible not just on the basis of its continued use for prolonged periods.

### Na_v_ 1.8 expression

After nerve injuries, the redistribution or expression of sodium channels can occur in primary nociceptive neurons, with a resulting low threshold for triggering pain or for the hyperexcitability of DRG neurons. The subtype Na_v_ 1.8 is selectively expressed in nociceptive, sensory neurons [2, 6, 35, 52], but to a greater extent in nerve pain models [15, 44] and also in patients with neuropathic pain [24, 50]. This is also thought to be important in both animals and humans for the spontaneous activity in neuromas [5, 24, 36], which might have played a role in our amputated patients (case report 2). This may also explain the reduction in attacks of pain in all our five patients concerned. Increased Na_v_ 1.8 expression also occurs in diabetic polyneuropathy [30], small-fibre neuropathy [19], radiculopathies [17, 44], trigeminal neuralgia [41], but also in arthritis [39, 43] and bone metastases [33]. Na_v_ 1.8 blockade (e.g. produced by ambroxol) is also ultimately considered as an option for the treatment of neuropathic pain, because investigations with Na_v_ 1.8-free mice and Na_v_ 1.8-blocking substances have shown lower mechanical, thermal and visceral hyperexcitability in the animal model [1, 4, 11, 18, 20, 21, 45, 46].

### Clinical significance, safety and dosage

Although ambroxol binds to the same local anaesthetic binding sites as lidocaine and amitriptyline, none of the substances used to date for neuropathic pain (includes local anaesthetics, antidepressants and anticonvulsants) have shown any relevant selectivity for the Na_v_ 1.8 channel comparable with that of ambroxol [27, 48]. The sodium-channel blockers used for treating pain, that is amitriptyline and carbamazepine, also do not block the channel types selectively [7, 42]. The selective blockade by ambroxol of the Na_v_ 1.8 channel type, which is not represented in the cardiac or central nervous systems, may therefore prove clinically beneficial. At least we did not receive any reports of corresponding adverse effects in any of our patients, including in those who have been using the substance topically for a long time now. This is also not surprising in that, despite the potent sodium channel blockade, the tolerability of ambroxol itself is very good after systemic administration: even intravenous doses as high as 1 g (for promoting prenatal lung maturation and the treatment of atelectasis) are well tolerated [51, 53]. There are even isolated reports of doses of up to 3 g a day being administered for up to 53 days [9, 28, 38] and the oral administration of 1.3 g ambroxol a day for 33 days [22]. After animal studies showed that high-dose ambroxol was clearly analgesic and well tolerated, Gaida et al. [14] presumed that these high dosages were probably necessary to produce analgesia. But with our topical treatments, we were able to show that even low-dose peripheral applications can have a clearly analgesic effect. Furthermore, no skin changes were observed by us, even though corresponding case reports exist [31].

In view of this interesting therapeutic approach, a number of other Na_v_ 1.8-blocking substances are currently being developed. However, its highly potent blockade, good bioavailability and very few side effects make ambroxol a particularly interesting substance, and one that should prove at least as useful as other sodium channel blockers for the treatment of chronic pain [27]. The safety and tolerability of systemically administered ambroxol—even at dosages on the gram scale—have been demonstrated over decades of clinical experience.

### Ambroxol and allodynia

Apart from case report 6, all patients suffered from hypersensibility to mechanical stimuli which regressed in each case after topical ambroxol. Is this anti-allodynic, analgesic effect really conceivable?

Allodynia and hyperalgesia are considered to be neuroplastic phenomena of the spinal sensory system [25]. The Na_v_ 1.8 channel is detected mainly in C- or A-delta fibres and neurons of the posterior horn [2, 6, 35, 52], although it is also expressed in A-beta fibres [1, 4, 26, 34, 40]. But since chronic inflammation shifts the excitability of Na_v_ 1.8 toward hyperpolarization this contributes to the allodynia, which means that ambroxol blockade would therefore be particularly pain-relieving. In animal studies, ambroxol also suppressed allodynia to differing degrees after systemic administration: heat hyperalgesia by 100 %, cold hyperalgesia and mechanical allodynia by approximately 75 % [14]. The intrathecal administration also showed an anti-allodynic effect in animal experiments [32]. The observation that ambroxol also managed to reduce mechanical allodynia in rats with experimental induced inflammation by approximately 2/3 [4] suggests that the anti-allodynic, analgesic effect is not necessarily limited just to neuropathic pain. In the patients described in this article, at least, the allodynia in response to mechanical stimuli was alleviated in all cases by topical ambroxol, which can now be explained by the rationale described above.

### Onset and duration of effect

The onset of Na_v_ 1.8 blockade in vitro by ambroxol starts within a few seconds and is concentration-dependent and fully reversible [27]. In paraplegic rats hypersensitivity to static mechanical stimuli was reduced after approximately 30 min for approximately 3 h [16]. Both findings correlate closely with the statements of our patients (Fig. 2), according to which the effect started between 15 and 30 min after application. In case report 1, the effect was frequently even said to appear ‘immediately’ and persisted for well over 6 h in this patient. When applied repeatedly, topical lidocaine 5 % often shows an increasing effect over time, both clinically and according to the literature [29]. A comparable result was also reported by our patient 6, who felt that his underlying pain had almost disappeared after 4 months of use. The significance of these observations needs to be explored in greater depth.

## Limitations

This case series describes (in an open-label, uncontrolled design) examples of the successful outcome of a topical treatment with ambroxol 20 % in patients with neuropathic pain. It is therefore still subject to numerous possible sources of error, from placebo effects to temporary therapeutic successes with no long-term significance, even though some of the treatments are documented here for several years. Although these were pooled, by way of example, for many other patients, they were compiled only retrospectively and, in this article, only for neuropathic pain. Since amount and depth of ambroxol’s skin penetration from the cream are not known, it cannot be stated conclusively whether the analgesic effects are based solely on its local action or on partial systemic effects. However, the latter would probably already have come to light clinically in view of the widespread use of ambroxol for respiratory disorders. An improvement to this initial formulation would also doubtless be possible. Therefore the observations do not yet allow any general conclusions to be drawn concerning the degree of efficacy in humans. Systematic and controlled studies on these questions are needed.

## Conclusion for practice

The expectorant ambroxol also acts as a very potent local anaesthetic. It significantly and preferentially blocks the nociceptively relevant sodium channel subtype Na_v_ 1.8 to a greater extent than all other local anaesthetics. Increased Na_v_ 1.8 expression is detected in neuropathic pain and is almost exclusively limited to sensory pain fibres. Its blockade is therefore considered to be a useful, simple concept for pain management with few side effects. In the case reports presented in this article, this concept was successfully implemented, for the first time, in the form of topical ambroxol 20 % cream in patients with severe neuropathic pain. This preparation has meanwhile been used for over 4 years without any undesirable effects. Since the development of new substances for the treatment of neuropathic pain is very arduous and time-consuming, we believe that there is an urgent need for further extensive research on the clinical use of well-known traditionally used substances with clear anti-nociceptive effects and positive side effect profiles, for example ambroxol. In view of the considerable distress caused by failed treatments the use of such substances should also be considered in connection with individual therapeutic trials.

### Contributions

The first author treated and documented the patients described in this paper, while the second author characterized the mechanism of action of ambroxol as a sodium channel blocker and investigated its effect in the preclinical pain models. The authors jointly drafted and critically discussed the manuscript. They would like to thank the pharmacist Martin Hofmann, Aukamm Apotheke Wiesbaden, for preparing the ambroxol cream formulation.

## References

[1] Abrahamsen B, Zhao J, Asante CO (2008). The cell and molecular basis of mechanical, cold, and inflammatory pain. Science.

[2] Akopian AN, Sivilotti L, Wood JN (1996). A tetrodotoxin-resistant voltage-gated sodium channel expressed by sensory neurons. Nature.

[3] Akopian AN, Souslova V, England S (1999). The tetrodotoxin-resistant sodium channel SNS has a specialized function in pain pathways. Nat Neurosci.

[4] Belkouch M, Dansereau MA, Tetreault P (2014). Functional up-regulation of Na_v_ 1.8 sodium channel in Abeta afferent fibers subjected to chronic peripheral inflammation. J Neuroinflammation.

[5] Black JA, Nikolajsen L, Kroner K (2008). Multiple sodium channel isoforms and mitogen-activated protein kinases are present in painful human neuromas. Ann Neurol.

[6] Blair NT, Bean BP (2002). Roles of tetrodotoxin (TTX)-sensitive Na+ current, TTX-resistant Na+ current, and Ca2+ current in the action potentials of nociceptive sensory neurons. J Neurosci.

[7] Brau ME, Dreimann M, Olschewski A (2001). Effect of drugs used for neuropathic pain management on tetrodotoxin-resistant Na(+) currents in rat sensory neurons. Anesthesiology.

[8] Catterall WA (2012). Voltage-gated sodium channels at 60: structure, function and pathophysiology. J Physiol.

[9] Chiara OBG, Padalino P, Guadalupi P, Bigaletto I, Nespoli A (1983). The prevention of postoperatíve prevention ARDS. Double-blind testin about the effects of ambroxol on pulmonary gas exchange. Urgenties Chirurgicae Commentaria.

[10] De Mey C, Peil H, Kolsch S (2008). Efficacy and safety of ambroxol lozenges in the treatment of acute uncomplicated sore throat. EBM-based clinical documentation. Arzneimittel-Forschung.

[11] Ekberg J, Jayamanne A, Vaughan CW (2006). muO-conotoxin MrVIB selectively blocks Na_v_ 1.8 sensory neuron specific sodium channels and chronic pain behavior without motor deficits. Proc Natl Acad Sci U S A.

[12] Farrar JT, Pritchett YL, Robinson M (2010). The clinical importance of changes in the 0–10 numeric rating scale for worst, least, and average pain intensity: analyses of data from clinical trials of duloxetine in pain disorders. J Pain.

[13] Ferrante FM, Paggioli J, Cherukuri S (1996). The analgesic response to intravenous lidocaine in the treatment of neuropathic pain. Anesth Analg.

[14] Gaida W, Klinder K, Arndt K (2005). Ambroxol, a Na_v_ 1.8-preferring Na(+) channel blocker, effectively suppresses pain symptoms in animal models of chronic, neuropathic and inflammatory pain. Neuropharmacology.

[15] Gold MS, Weinreich D, Kim CS (2003). Redistribution of Na(V)1.8 in uninjured axons enables neuropathic pain. J Neurosci.

[16] Hama AT, Plum AW, Sagen J (2010). Antinociceptive effect of ambroxol in rats with neuropathic spinal cord injury pain. Pharmacol Biochem Behav.

[17] He XH, Zang Y, Chen X (2010). TNF-alpha contributes to up-regulation of Na_v_ 1.3 and Na_v_ 1.8 in DRG neurons following motor fiber injury. Pain.

[18] Hillsley K, Lin JH, Stanisz A (2006). Dissecting the role of sodium currents in visceral sensory neurons in a model of chronic hyperexcitability using Na_v_ 1.8 and Na_v_ 1.9 null mice. J Physiol.

[19] Huang J, Yang Y, Zhao P (2013). Small-fiber neuropathy Na_v_ 1.8 mutation shifts activation to hyperpolarized potentials and increases excitability of dorsal root ganglion neurons. J Neurosci.

[20] Jarvis MF, Honore P, Shieh CC (2007). A-803467, a potent and selective Na_v_ 1.8 sodium channel blocker, attenuates neuropathic and inflammatory pain in the rat. Proc Natl Acad Sci U S A.

[21] Joshi SK, Mikusa JP, Hernandez G (2006). Involvement of the TTX-resistant sodium channel Na_v_ 1.8 in inflammatory and neuropathic, but not post-operative, pain states. Pain.

[22] Kimya Y, Kucukkomurcu S, Ozan H (1995). Antenatal ambroxol usage in the prevention of infant respiratory distress syndrome. Beneficial and adverse effects. Clin Exp Obstet Gynecol.

[23] Klier KF, Papendick U (1977). The local anesthetic effect of NA872-containing eyedrops. Med Monatsschr.

[24] Kretschmer T, Happel LT, England JD (2002). Accumulation of PN1 and PN3 sodium channels in painful human neuroma-evidence from immunocytochemistry. Acta Neurochir.

[25] Kuner R (2010). Central mechanisms of pathological pain. Nat Med.

[26] Lai J, Gold MS, Kim CS (2002). Inhibition of neuropathic pain by decreased expression of the tetrodotoxin-resistant sodium channel, Na_v_ 1.8. Pain.

[27] Leffler A, Reckzeh J, Nau C (2010). Block of sensory neuronal Na+ channels by the secreolytic ambroxol is associated with an interaction with local anesthetic binding sites. Eur J Pharmacol.

[28] Luerti M, Lazzarin A, Corbella E (1987). An alternative to steroids for prevention of respiratory distress syndrome (RDS): multicenter controlled study to compare ambroxol and betamethasone. J Perinat Med.

[29] Meier T, Wasner G, Faust M (2003). Efficacy of lidocaine patch 5 % in the treatment of focal peripheral neuropathic pain syndromes: a randomized, double-blind, placebo-controlled study. Pain.

[30] Mert T, Gunes Y (2012). Antinociceptive activities of lidocaine and the Na_v_ 1.8 blocker a803467 in diabetic rats. J Am Assoc Lab Anim Sci.

[31] Monzon S, Del Mar Garces M, Lezaun A (2009). Ambroxol-induced systemic contact dermatitis confirmed by positive patch test. Allergol Immunopathol.

[32] Moon JY, Song S, Yoon SY (2012). The differential effect of intrathecal Na_v_ 1.8 blockers on the induction and maintenance of capsaicin- and peripheral ischemia-induced mechanical allodynia and thermal hyperalgesia. Anesth Analg.

[33] Qiu F, Jiang Y, Zhang H (2012). Increased expression of tetrodotoxin-resistant sodium channels Na_v_ 1.8 and Na_v_ 1.9 within dorsal root ganglia in a rat model of bone cancer pain. Neurosci Lett.

[34] Renganathan M, Cummins TR, Hormuzdiar WN (2000). Alpha-SNS produces the slow TTX-resistant sodium current in large cutaneous afferent DRG neurons. J Neurophysiol.

[35] Renganathan M, Cummins TR, Waxman SG (2001). Contribution of Na(v)1.8 sodium channels to action potential electrogenesis in DRG neurons. J Neurophysiol.

[36] Roza C, Laird JM, Souslova V (2003). The tetrodotoxin-resistant Na+ channel Na_v_ 1.8 is essential for the expression of spontaneous activity in damaged sensory axons of mice. J Physiol.

[37] Rush AM, Cummins TR, Waxman SG (2007). Multiple sodium channels and their roles in electrogenesis within dorsal root ganglion neurons. J Physiol.

[38] Schillings GJPJ (1992). Ambroxol in the treatment of ARDS in polytraumatised patients -a report. Klinikarzt.

[39] Schuelert N, Mcdougall JJ (2012). Involvement of Na_v_ 1.8 sodium ion channels in the transduction of mechanical pain in a rodent model of osteoarthritis. Arthritis Res Ther.

[40] Shields SD, Ahn HS, Yang Y (2012). Na_v_ 1.8 expression is not restricted to nociceptors in mouse peripheral nervous system. Pain.

[41] Siqueira SR, Alves B, Malpartida HM (2009). Abnormal expression of voltage-gated sodium channels Na_v_ 1.7, Na_v_ 1.3 and Na_v_ 1.8 in trigeminal neuralgia. Neuroscience.

[42] Song JH, Ham SS, Shin YK (2000). Amitriptyline modulation of Na(+) channels in rat dorsal root ganglion neurons. Eur J Pharmacol.

[43] Strickland IT, Martindale JC, Woodhams PL (2008). Changes in the expression of Na_v_ 1.7, Na_v_ 1.8 and Na_v_ 1.9 in a distinct population of dorsal root ganglia innervating the rat knee joint in a model of chronic inflammatory joint pain. Eur J Pain.

[44] Thakor DK, Lin A, Matsuka Y (2009). Increased peripheral nerve excitability and local Na_v_ 1.8 mRNA up-regulation in painful neuropathy. Mol Pain.

[45] Veneroni O, Maj R, Calabresi M (2003). Anti-allodynic effect of NW-1029, a novel Na(+) channel blocker, in experimental animal models of inflammatory and neuropathic pain. Pain.

[46] Villarreal CF, Sachs D, Cunha FQ (2005). The role of Na(V)1.8 sodium channel in the maintenance of chronic inflammatory hypernociception. Neurosci Lett.

[47] Weiser T (2006). Comparison of the effects of four Na+ channel analgesics on TTX-resistant Na+ currents in rat sensory neurons and recombinant Na_v_ 1.2 channels. Neurosci Lett.

[48] Weiser T (2006). Na_v_ 1.8 channel blockade as an approach to the treatment of neuropathic pain. Drugs Fut.

[49] Weiser T, Wilson N (2002). Inhibition of tetrodotoxin (TTX)-resistant and TTX-sensitive neuronal Na(+) channels by the secretolytic ambroxol. Mol Pharmacol.

[50] Yiangou Y, Birch R, Sangameswaran L (2000). SNS/PN3 and SNS2/NaN sodium channel-like immunoreactivity in human adult and neonate injured sensory nerves. FEBS Lett.

[51] Zhang ZQ, Wu QQ, Huang XM (2013). Prevention of respiratory distress syndrome in preterm infants by antenatal ambroxol: a meta-analysis of randomized controlled trials. Am J Perinatol.

[52] Zimmermann K, Leffler A, Babes A (2007). Sensory neuron sodium channel Na_v_ 1.8 is essential for pain at low temperatures. Nature.

[53] Zwissler B (2002). Pharmakotherapie bei akutem Lungenversagen. J Anaesth Intensivbehandl.

